# Unmet clinical needs in women with polycystic ovary syndrome regarding chronic non-communicable diseases: A cross‑sectional study

**DOI:** 10.1007/s00404-025-08287-x

**Published:** 2026-01-08

**Authors:** Susanne Theis, Elena Pavicic, Julia Estermann, Norman Bitterlich, Hamideh Frühwein, Petra Stute

**Affiliations:** 1 Department of Obstetrics and Gynecology, University Medical Center of Johannes Gutenberg University Mainz, Langenbeckstraße 1, 55131 Mainz, Germany; 2 Division of Gynaecological Endocrinology and Reproductive Medicine, Department of Gynaecology and Obstetrics, University Hospital Inselspital Bern, Bern, Switzerland; 3 Faculty of Medicine, University of Bern, Bern, Switzerland; 4 Freelance Statistician, Chemnitz, Germany; 5 Institute for History and Ethics University Medical Center, Johannes Gutenberg University Mainz, Mainz, Germany; 6 Department of Obstetrics and Gynaecology, University Hospital Inselspital, Friedbuehlstrasse 19, 3010 Bern, Switzerland

**Keywords:** Polycystic ovarian syndrome, Non- communicable diseases, Risk assessment, Screening, Patient satisfaction

## Abstract

**Purpose:**

Polycystic ovary syndrome (PCOS) is recognised as a potential risk factor for chronic non communicable diseases (NCD). Although international guidelines recommend proactive NCD risk prevention, actual practice may be suboptimal. This study aimed to identify unmet clinical needs regarding information, risk assessment and satisfaction with care related to NCD risk factors among women with PCOS.

**Methods:**

An eight-domain questionnaire was developed based on the 2018 ESHRE guideline, covering demographics, PCOS diagnostic criteria, aesthetics, metabolism, reproduction, mental health and NCD prevention /monitoring. The present analysis focused on metabolic disorders, guideline-recommended risk screenings, patients’ satisfaction with care and overall satisfaction with management by healthcare providers (HCPs).

**Results:**

Of 2029 respondents, 1943 answered metabolic-related items. 66.3% without known metabolic disease (MD) reported never having undergone screening for MD. 34.3% received advice from gynaecologist, 58.9% from general practitioner (GP) concerning MD. 41.1% (n = 271) did not receive counselling. Among 1839 respondents, 32.5% reported gynaecologist-led risk discussions. Annual screening occurred in 30.5% (body weight), 46.8% (blood pressure), 5.8% (gynaecologist) to 21.4 (GP) for serum cholesterol and 25.4% for diabetes. 1.0% had been consulted on obstructive sleep apnoea (OSA), 17.5% on endometrial cancer. Satisfaction with gynaecologist counselling was low (Mean 34.7), 79.4% expressed a desire for more advice.

**Conclusion:**

Women with PCOS remain at high risk for NCDs (CVD, diabetes, endometrial cancer, OSA), yet experience substantial gaps in risk awareness, monitoring, and counseling. Addressing these deficiencies through improved clinical practice, education and adopting holistic PCOS management that balances NCD prevention with infertility concerns, is essential for safeguarding long-term health.

**Supplementary Information:**

The online version contains supplementary material available at 10.1007/s00404-025-08287-x.

## Introduction

Polycystic ovary syndrome (PCOS) is the most common endocrine disorder, among women of reproductive age [1]. Its prevalence varies depending on diagnostic criteria used and is estimated at 7–13%, with a considerable proportion of cases likely remaining undiagnosed [2, 3]. Diagnostic features of PCOS include oligomenorrhoea (cycle length > 35 days) or amenorrhoea (absence of menstrual periods), clinical hyperandrogenism (e.g. hirsutism, acne, alopecia) and/or hyperandrogenaemia (elevated levels of free testosterone, free androgen index, or bioavailable testosterone in blood), and polycystic ovarian morphology (≥ 20 per ovary and/or an ovarian volume ≥ 20 ml). PCOS is also associated with several comorbidities including an increased risk for chronic non-communicable diseases (NCD) such as cardiovascular disease (CVD), insulin resistance, type 2 diabetes mellitus (T2DM) and mental health conditions including depression, anxiety, and eating disorders [4–6]. Consequently, PCOS requires a holistic, interdisciplinary management approach.

The international, evidence-based guideline published by the European Society of Human Reproduction and Embryology (ESHRE) serves as a framework for the diagnosis, management, and follow-up of women with PCOS [7]. According to the 2018 ESHRE guidelines (valid at the time of data collection), recommended screenings include weight monitoring, blood pressure, glycaemic status, cardiovascular risk, fasting lipid profile, oral glucose tolerance test (OGTT), screening for obstructive sleep apnoea (OSA), and evaluation of emotional well-being. A 2023 revision introduced minor updates to these recommendations. (Table S_1) [8].

For NCD risk screening, regular measurements of weight, cholesterol, and blood sugar measurements, as well as OGTT prior to pregnancy, are recommended. However, it remains unclear to what extent healthcare providers (HCP) implement these recommendations in routine practice. Indeed, previous studies have reported a considerable patient dissatisfaction with care received form HCP [8–12].

The national, evidence-based guideline published by the Association of the Scientific Medical Societies in Germany (AWMF) [13] defines polycystic ovary syndrome (PCOS) according to the modified Rotterdam criteria. The diagnosis should be established when at least two of the following three features are present: clinical and/or biochemical hyperandrogenism, ovulatory dysfunction, and polycystic ovarian morphology (PCOM) and/or elevated anti-Müllerian hormone (AMH) concentrations, after exclusion of relevant differential diagnoses.

The national guideline recommends – similar to the ESHRE guideline: women with PCOS should receive structured counseling on their increased risk for metabolic syndrome and cardiovascular disease, liver disorders, sleep-related breathing disorders, endometrial cancer, Hashimoto’s thyroiditis, depression, eating disorders, and reduced quality of life, along with evidence-based strategies for prevention, monitoring and management of these comorbidities.

The aim of this cross-sectional cohort study was therefore to evaluate the unmet clinical needs and patient-reported satisfaction with healthcare management provided to women with PCOS. We hypothesized that many women do not receive counselling in line with the ESHRE and national guidelines. Instead they seek comprehensive care, including management of hyperandrogenism, metabolic issues, menstrual irregularities, fertility, mental health, and NCD prevention. A strong correlation between NCD and PCOS is well established [14, 15]. In this publication, we focus on NCD prevention, given the growing evidence supporting PCOS as an independent risk factor for cardiovascular morbidity [16].

## Materials and methods

This cross-sectional cohort study, conducted via an online survey, included German- speaking women aged > 18 years, who either met the ESHRE diagnostic criteria for PCOS [7, 8], or had been diagnosed with PCOS by a gynaecologist. Women were excluded if postmenopausal or had been diagnosed with other causes of hyperandrogenism. Recruitment took place between January and March 2021.

A recruitment flyer containing the link to the survey (Supplementary file 1) was distributed through multiple online channels. To prevent double entries, participants created a personal identification code.

The Cantonal Ethics Committee of Bern (Req-2020–00801) confirmed that no ethical approval was required (Supplementary file 2) for this study.

This publication focusses on the topic of NCD screening and prevention in women with PCOS. Findings from other domains have been published previously [11, 12, 17].

### Questionnaire

An eight-domain questionnaire (Supplementary File 3), previously described in detail [11, 12], was developed through a multi-step process. This included testing by 15 volunteers, statistical evaluation, and final review by the principal investigator. The questionnaire covered all domains outlined in the ESHRE guidelines [8], namely demographics, PCOS diagnostic criteria, aesthetics, metabolism, menstrual cycle, reproduction, mental health, and NCD prevention and monitoring.

The NCD-related items addressed body image, eating patterns, and emotional wellbeing. Screening questions were adapted from the 2018 ESHRE guideline for PCOS (Table_S_1). For this subgroup analysis, all women who answered at least one question regarding metabolic disorders (MD) or risk monitoring were included. Questions on metabolic disorders focused on recommended screenings for diabetes and related conditions, particularly during pregnancy. Questions on risk monitoring assessed whether recommended screenings for NCDs in women with PCOS had been performed, covering conditions such as CVD, DM, OSA, and endometrial cancer.

Participants were asked to rate their overall satisfaction with their HCPs’ management of metabolic disease and NCD risk monitoring, and to indicate whether they desired more detailed consultations or specific information during medical encounters.

To minimise information bias, questions were phrased neutrally, participants blinded to hypotheses, medical terminology explained, input validation was implemented, and the option to select “unknown” was provided. The questionnaire was programmed and administered using REDCap software to ensure secure data management.

### Statistical analysis

A sample size of 199 participants was required based on power calculations using the G*Power Software, with satisfaction measured on a scale from 0 to 100 as the parameter of interest. The calculation aimed to detect a minimum difference of 10% in observed frequencies from an expected frequency of 50%, which requires the largest sample size. Recruitment, however, was not halted after 199, continuing for 10 weeks.

Descriptive statistics were used to characterise the study population. Subgroup analyses were conducted for each risk monitoring domain. Descriptive values such as mean (with standard deviation), median, and appropriate statistical tests were applied, including the Mann–Whitney U test, Chi-square test, and Jonckheere–Terpstra test. A p-value of < 0.05 was considered statistically significant. Effects of multiple testing were not taken into account in this exploratory analysis. Data were analysed using SPSS version 27.0.

## Results

### Characteristics of the cohort

**Fig. 1:**
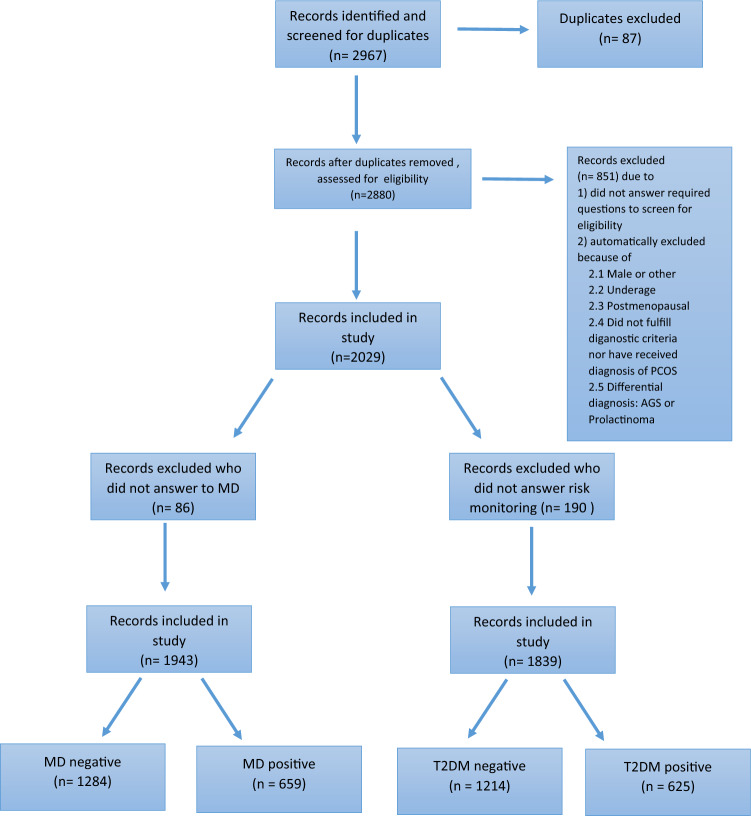
Record Screening Process, Abbreviations: n = number, MD = metabolic disorder, DM = Type 2 Diabetes mellitus

A total of 2967 survey responses were received. After removing duplicate entries and applying eligibility criteria, 2029 participants were included in the final analysis (Fig. 1). Of these, 1720 women (84.8%) reported having received a PCOS diagnosis from their gynaecologist, while the remainder fulfilled PCOS diagnostic criteria without a formal diagnosis.

All participants were female, 72.9% lived in Germany, 70.3% were childless, 60.7% held a university degree and 63.8% were employed at least part-time. The mean age was 28.9 ± 5.5 years, (median: 29 years). The mean BMI was 30.5 ± 8.5 kg/m2 (median: 29.8 kg/m2), with 49.8% of the participants classified as obese.

Of the 2029 respondents, 86 women did not answer the question about metabolic disorder (MD). These were excluded from the analysis, leaving 1943 (95.8%) individuals who provided at least one answer concerning MD. Among these, 659 participants (33.9%) reported that they were suffering from MD. 1839 participants provided information on at least one risk monitoring subgroup, including 625 individuals who had also reported a diagnosis of DM.

### Metabolic disorder

Among the 1943 participants who provided valid responses, 659 women (33.9%) reported having a diagnosed metabolic disorder. Of these, 70 participants indicated DM (11 with T1DM, 51 with T2DM), 123 reported gestational diabetes and 466 pre-diabetes. In cases of multiple responses, participants were assigned to the highest applicable diagnostic category.

Among participants with DM (T1DM and T2DM), 95.7% reported at least one form of glucose screening. This rate was similarly high in the gestational diabetes group (97.5%) and in the prediabetes group (95%). In contrast, only 66.3% of participants without a diagnosed metabolic disorder reported a blood glucose test.

Regarding counselling, 34.3% of affected participants reported receiving advice from their gynaecologist, and 58.9% from their general practitioner (GP). Counselling rates varied significantly by condition: among those with DM, 87.1% received advice from a GP and 35.7% from a gynaecologist; in the gestational diabetes group, 75.6% were advised by a GP and 49.6% by a gynaecologist; in the prediabetes group, 50.2% received advice from a GP and 30.0% from a gynaecologist.

Although differences in satisfaction with gynaecological counselling across the three groups were not statistically significant (*p* = 0.107), the proportion of participants desiring more advice differed significantly (*p* < 0.001): 51.1% in the DM group, 59.7% in the gestational diabetes group, and 74.8% in the prediabetes group.

Overall, 41.1% (n = 271) of affected women reported receiving no counselling from either a GP or a gynecologist.

### Risk assessment

In the present cohort, we evaluated patient-reported information, risk assessment and satisfaction regarding four key risk factors associated with PCOS: CVD, DM, OSA and endometrial cancer.

Overall, 32.5% of participants (n = 1839) reported that their gynaecologist had discussed potential health risks related to PCOS. Looking specifically at the subgroup of participants with DMs (n = 625), 37.8% affirmed the statement that they were informed about potentially increased risks due to PCOS and 29.8% stated “no DM” (n = 1214, *p* < 0.001). The risks addressed during these discussions included CVD (16%), DM (26.5%), OSA (2.3%) and endometrial cancer (9.8%). An additional 2.2% reported being informed about “other” risks, most commonly infertility.

Self-reported cardiovascular risk factors included smoking, obesity, elevated cholesterol, elevated blood pressure, impaired glucose tolerance, lack of exercise, and a family history of myocardial infarction or stroke. All these risk factors occurred significantly more frequently among women with DM compared to those without (Table 1).

**Table 1:** Self-reported cardiovascular risk factors by diabetes status

Risk factor	Total share in %	Share without DM in %	Share with DM in %	P – value
Smoking	21.0 (19.1–23.0)	18.9 (16.7–21.3)	25.0 (21.6–28.6)	0.003
Obesity	65.2 (62.9–67.4)	54.0 (51.1–56.9)	86.9 (83.9–89.5)	< 0.001
Elevated Cholesterol	10.6 (9.2–12.2)	8.6 (7.1–10.4)	14.4 (11.7–17.5)	< 0.001
Elevated blood pressure	13.1 (11.5–14.8)	8.0 (6.5–9.7)	23.0 (19.7–26.6)	< 0.001
Impaired sugar tolerance	34.0 (31.8–36.3)	9.7 (8.1–11.6)	81.3 (77.9–84.3)	< 0.001
Lack of exercise	31.4 (29.3–33.7)	27.2 (24.6–29.8)	39.7 (35.8–43.7)	< 0.001
heart attack/ stroke in family history	26.4 (24.3–28.5)	23.9 (21.5–26.4)	31.2 (27.5–35.0)	0.001

DM = diabetes mellitus

In total, 8.5% of participants reported a current or past history of CVD. More detailed information on disease and frequency can be found in Table 2.

**Table 2:** Self-reported history of cardiovascular disease by diabetes status

Disease	Total share in %	Share without DM in %	Share with DM in %	P – value
Elevated Blood pressure	6.1 (5.0–7.4)	3.0 (2.0–4.1)	12.3 (9.8–15.2)	< 0.001
Heart attack	0.0 (0.0 –0.1)	0.0 (0.0–0.3)	0.0 (0.0–0.5)	-
Stroke	0.1 (0.0–0.4)	0.2 (0.0–0.8)	0.0 (0.0–0.5)	0.310
Lung embolia	0.3 (0.0–0.7)	0.2 (0.0–0.8)	0.3 (0.0–1.4)	0.776
Thrombosis	0.8 (0.4–1.3)	0.7 (0.2–1.3)	1.0 (0.3–2.1)	0.482

DM = diabetes mellitus

#### Risk assessment cardio- vascular disease

Indicated screenings for cardiovascular risk factors are summarized in Table 3. Notably, 80.7% of the participants reported not receiving regular cholesterol checks. It should be noted, however, that responses regarding annual cholesterol checks by gynaecologist or GPs were incomplete (n = 1539 and n = 1550).

**Table 3:** Annual screening measures reported by participants

Screening annually	Total share in %	Share without DM in %	Share with DM in %	P – value
Weight by gynaecologist	30.5 (28.4–32.7)	31.1 (28.4–33.8)	29.5 (25.9–33.3)	0.490
Blood pressure by gynaecologist	46.8 (44.4–49.1)	46.5 (43.6–49.4)	47.4 (43.4–51.5)	0.691
Cholesterol by gynaecologist*	5.8 (4.6–7.1)	5.8 (4.4–7.4)	5.8 (3.9–8.2)	0.978
Cholesterol by GP **	21.4 (19.4–23.6)	17.1 (14.8–19.6)	29.6 (25.7–33.7)	< 0.001

##### Advice about increased cardiovascular risks and satisfaction with advice

Overall, 10.7% of the participants (no DM 9.1%, with DM 13.8%, *p* = 0.002) stated being informed by their gynaecologist about elevated risks of CVD associated with PCOS. Satisfaction was measured on a scale from 0 (not satisfied at all) to 100 (highest satisfaction). Satisfaction due to counselling did not differ significantly between the different subgroups.

Among those who had not received advice, 80.2% indicated they would have wished for such counselling. This proportion differed significantly between the subgroups (no DM: 80.2%; with DM: 85.9%; *p* = 0.005).

Mean satisfaction scores for CVD risk counselling (scale 0–100) were as follows: total cohort- 67.0 (median: 67.5; SD: 26.5), without DM 69.7 (median: 74.5; SD: 26.7) and with DM- 63.5 (median: 62.5; SD: 25.9). The difference in satisfaction between the groups was not statistically significant (p = 0.075).

#### Risk assessment diabetes

Annual blood glucose testing by a gynaecologist was reported by 11.3% of the participants (9.8% without DM vs. 14.2% with DM, p = 0.005). Annual blood glucose testing by a GP or endocrinologist was reported by 25.4% of the participants ((15.5% without DM vs. 44.4% with DM; *p* < 0.001). Overall, only 25.4% of the participants reported receiving annual diabetes screening.

Recommended OGTT before pregnancy was performed in 34.3% of the respondents (no DM 25.9%, with DM 45.8%, p = 0.02). OGTT during pregnancy was reported by 21.0% (no DM 18.8%, with DM 24.1%, p = 0.44).

##### Advice about increased DM risk and satisfaction with advice

In total, 33.3% stated they were informed about elevated risk of DM relate to PCOS (without DM 28.5% vs. with DM 42.5%, *p* < 0.001). Satisfaction with counselling did not differ significantly between the groups.

Among those who had not received advice, 79.3% indicated they would have wished for such information. This desire was significantly more frequent among the subgroups (no DM 76.8% and 85.2% with DM, p = 0.001).

Mean satisfaction scores for DM risk counselling (scale 0–100) were as follows: total cohort-58.5 (median: 57.0; SD: 28.0), without DM- 58.4 (median: 60.0; SD: 29.0) and with DM- 58.7 (median: 52.0; SD: 26.8). There was no significant difference in satisfaction between groups (p = 0.904).

#### Risk assessment obstructive sleep apnea disorder (OSA)

Symptoms of OSA were assessed based on participant self-report and are summarised in Table 4. These included annual cholesterol testing by a GP (n = 1550), snoring, observed episodes of breathing interruption during sleep, unrefreshing sleep, daytime sleepiness, morning headaches, and concentration problems. All of these risk factors were reported significantly more often by participants with diabetes compared to those without.

**Table 4:** OSA-related symptoms and testing by diabetes status

Risk factor	Total share in %	Share without DM in %	Share with DM in %	P – value
Annually checking cholesterol by GP	21.4 (19.4–23.6)	17.1 (14.8–19.6)	29.6 (25.7–33.7)	< 0.001
Snoring	36.1 (33.9–38.4)	31.1 (28.4–33.8)	45.9 (41.9–50.0)	< 0.001
Interrupted breathing during sleep (e.g. observed by partner)	7.5 (6.3–8.9)	5.4 (4.1–6.8)	11.7 (9.2–14.5)	< 0.001
Awakening unrefreshed	52.6 (50.3–55.0)	48.5 (45.6–51.4)	60.6 (56.6–64.5)	< 0.001
Daily sleepiness	48.6 (46.3–51.0)	44.6 (41.8–47.5)	56.3 (52.3–60.3)	< 0.001
Headache in the morning	29.5 (27.4–31.7)	27.3 (24.8–30.0)	33.8 (30.0–37.7)	0.004
Concentration disorders	43.7 (41.4–46.1)	41.4 (38.5–44.2)	48.3 (44.3–52.4)	0.004
None of them	20.1 (18.2–22.0)	24.1 (21.7–26.7)	12.2 (9.7–15.0)	< 0.001

DM = diabetes mellitus, GP = general practitioner

##### Advice about increased OSA risk and satisfaction with advice

Overall, 3.0% of the respondents (no DM 2.2%, with DM 4.5%, p = 0.007) reported a known OSAS. In addition, 5.5% had been to a specialist for OSAS or a sleeping lab. Only 1.0% (no DM 1.2%, with DM 0.8% p = 0.478) stated they were advised by their gynaecologist of OSA risk in the context of PCOS.

Satisfaction with counselling did not differ significantly between the groups. Those who were not advised would have wished for advice in 54.0% (no DM 52.0% and 58.0% with DM (p = 0.015). Mean satisfaction scores for OSA counselling (scale 0–100) were as follows: total cohort -50.0 (median: 41.3; SD: 34.1), without DM- 22.5 (median: 32.5; SD: 34.0), with DM- 50.0 (median: 65.8; SD: 21.8). Satisfaction did not differ significantly between groups (p = 0.055).

#### Risk assessment endometrial cancer

A diagnosis of endometrial cancer was reported by 1.0% of participants (0.7% without DM vs. 1.4% with DM; p = 0.149).

##### Advice about increased endometrial cancer risk and satisfaction with advice

17.5% of particpants stated being advised by their gynaecologist about the risk of endometrial cancer associated with PCOS (17.0% without DM vs. 18.6% with DM; p = 0.391).

Mean satisfaction scores for endometrial cancer risk counselling (scale 0–100) were as follows: total cohort- 65.5 (median: 65.0; SD: 25.7), without DM- 67.0 (median: 69.0; SD: 25.0) and with DM- 61.3 (median: 60.5; SD: 26.7). Satisfaction levels did not differ significantly between the groups (p = 0.064).

Among participants who had not received counselling, 78.5% indicated they would have appreciated advice about this risk (77.4% without DM vs. 80.9% with DM; p = 0.112).

### Overall satisfaction with the care provided by the gynaecologist with regard to risk monitoring

Overall, satisfaction with counselling on PCOS-related risk factors provided by gynaecologists was low. The mean satisfaction score (scale 0–100) in the total cohort was 34.7 (median: 30.0; SD: 28.2), with 35.5 (median: 32.0; SD: 29.3) among participants without DM and 33.2 (median: 27.0; SD: 28.1) among those with DM. There was no statistically significant difference in satisfaction between the groups (p = 0.082).

A large proportion of participants (79.4%) expressed a wish for more comprehensive counselling, with slightly higher demand among women with diabetes (82.2%) compared to those without (78.0%; p = 0.034).

Regarding the type of counselling desired, significant differences were observed between groups (see Table 5):

**Table 5:** Types of additional counselling and support participants would have preferred

Type of advice desired	Total share in %	Share without DM in %	Share with DM in %	P – value
More advise and reassurance	71.4 (69.2–73.5)	69.4 (66.7–72.1)	75.2 (71.6–78.6)	0.010
More information (like brochures)	50.4 (48.0–52.7)	49.3 (46.4–52.2)	52.5 (48.4–56.5)	0.191
More space for questions	43.0 (40.7–45.4)	40.1 (37.3–43.0)	48.6 (44.6–52.7)	< 0.001
More testings (e.g. blood pressure, sugar, weight)	57.7 (55.3–60.0)	54.5 (51.6–57.4)	63.8 (59.9–67.7)	< 0.001
More therapeutical options	49.3 (47.0–51.7)	44.8 (41.9–47.7)	58.1 (54.1–62.0)	< 0.001

DM = diabetes mellitus

## Discussion

This cross-sectional online survey supported our hypothesis that women with PCOS are not sufficiently counselled in line with ESHRE guidelines regarding diagnosis treatment and follow-up. Although the guidelines provide clear, evidence-based recommendations, our findings reveal substantial gaps both in risk monitoring and patient education.

Overall, only 32.5% of participants confirmed that their gynaecologist had informed them about potentially increased risks due to their PCOS diagnosis, leaving 67.5% without relevant counselling. Consultation rates were higher among participants with DM—suggesting greater awareness of concomitant conditions in this subgroup (advice about risks with DM in 37.8% and without DM in 29.8%, *p* < 0.001)—yet this still reflects a considerable information deficit. Notably, 66.3% of women without a known metabolic disorder had never undergone a glucose test. This suggests a potentially higher number of undetected cases and therefore unrecognized long-term risks.

Gaps were evident across all guideline-recommended risk assessments: CVD (16%), DM (26.5%), OSA (2.3%) and endometrial cancer (9.8%). A closer look at the individual risk assessments showed that a screening occurred more likely when DM was already diagnosed. These findings underscore a sobering reality. PCOS patients are still not sufficiently identified as a high-risk population by GPs and gynaecologists and are therefore too rarely given preventive strategies. Similar patterns have been reported in other studies [18].

From the HPC perspective, there is a clear need for education and improved care structures to enable holistic, guideline-based PCOS management. Equally, there is significant room for improvement in patient education. The high percentage of participants expressing a desire for more advice (74.8%) suggests that those affected are highly motivated and willing to engage with preventive strategies. Comparable findings have been reported in Canada: patients expressed a need for greater knowledge and awareness of PCOS among HCPs, better access to specialist care and improved patient information resources [12, 19]. Furthermore, a recent meta-analysis on clinical CVD in PCOS emphasized the importance of comprehensive risk assessment in PCOS to reduce the long-term burden in high-risk population [16].

Beyond individual health outcomes, the broader economic and societal implications of undiagnosed or poorly managed PCOS should not be underestimated. Existing studies have examined the economic burden of PCOS, albeit only in relation to individual aspects: in the United States, estimated annual costs related to pregnancy complications and long-term morbidities totalled $4.3 billion USD in 2020 [20], mental health comorbidities (anxiety, depression, and eating disorders): $4.261 billion USD in 2021 [21], and endometrial cancer: $467 million USD in 2023 [22]. In German-speaking countries, diagnostic and therapeutic measures for PCOS are generally reimbursed when medically indicated. However, differences in insurance structures and regional regulations may influence access and continuity of care. Moreover, limited consultation time, reimbursement uncertainty and—in some cases—insufficient awareness or knowledge among attending gynaecologists or general practitioners may contribute to inadequate patient counselling and information provision [18]. Considering the untapped potential for early detection and prevention identified in this study, the findings underscore not only a personal health imperative but also a societal responsibility to act.

### Strength and weaknesses of the study

The survey was distributed via online channels to women with PCOS in German-speaking countries. Although the sample included women from various backgrounds, a selection bias due to online recruitment cannot be excluded. Despite outreach through universities, women with tertiary education were not overrepresented. The study focused on women’s subjective perspectives and therefore does not include the perspectives of HCP. All results and interpretations are based on self-reported data and rely on the assumption of truthful responses. As most participants were Caucasian, other ethnicities were underrepresented in the cohort.

A major strength of this study is its large sample size, which supports the representativeness of the findings. Most previous studies on PCOS have concentrated on infertility, often neglecting other key aspects of the condition [23–25]. In contrast, this study collected data on all main areas of PCOS. In conclusion, the high response rate underscores that women affected are seeking greater recognition and awareness of their condition. The overall findings highlight the clear deficits in healthcare services regarding risk assessment and preventive care for women with PCOS.

## Conclusion

The implementation of risk counselling and risk assessment for women with PCOS was found to be low, and satisfaction with provided management ranged from low to moderate. It is imperative for HCPs to be vigilant in screening for accompanying diseases and to provide appropriate counselling. Education on the disease and its implications for NCDs should be given equal emphasis as infertility counselling. A holistic approach to PCOS management is not only warranted, but strongly desired to ensure the well-being and overall health of women with PCOS. It is essential to improving both individual health outcomes and the long-term burden on healthcare systems.

## Supplementary Information

Below is the link to the electronic supplementary material.

Supplementary file1 (PDF 590 kb)

Supplementary file2 (PDF 60 kb)

Supplementary file3 (PDF 346 kb)

Supplementary file4 (DOCX 15 kb)

## Data Availability

No datasets were generated or analysed during the current study.

## Declarations

### What does this study add to the clinical work?

This study highlights the need for regular risk assessments and improved patient education for women with PCOS, who show a strong willingness to engage in their care. It also calls for greater awareness among healthcare professionals to ensure comprehensive, guideline-based management and improved long-term outcomes.
