# Supplementary material for: Unmet clinical needs in women with polycystic ovary syndrome regarding chronic non-communicable diseases: A cross‑sectional study
Source: Arch Gynecol Obstet. 2026 Jan 8;313(1):21. doi: 10.1007/s00404-025-08287-x (PMC12783285; doi:10.1007/s00404-025-08287-x)
Supplement: Supplementary file 1 — Supplementary file1 (PDF 590 kb) [file 404_2025_8287_MOESM1_ESM.pdf]

PCOS

Polyzystisches Ovarialsyndrom

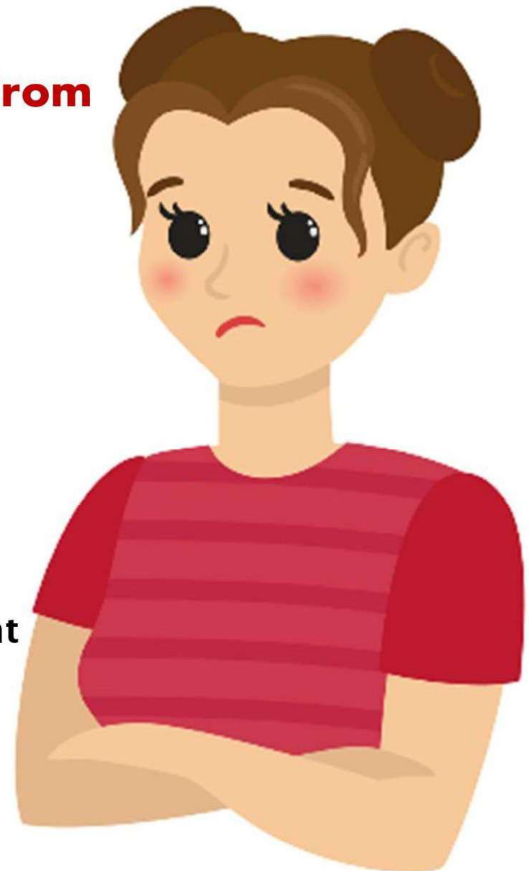

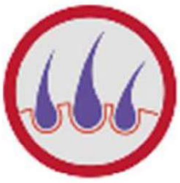

übermässige Körperbehaarung

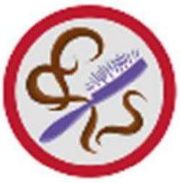

Haarausfall

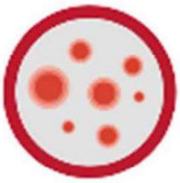

Akne

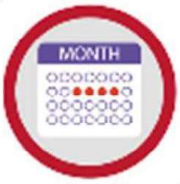

unregelmässige Menstruation

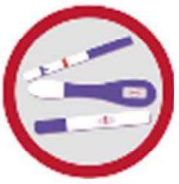

unerfüllter Kinderwunsch

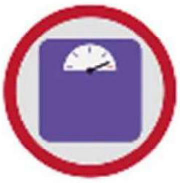

Übergewicht

Treffen diese Symptome auf Sie zu oder ist bei Ihnen PCOS bekannt?

Dann sind Sie hier genau richtig!

STUDIEN-TEILNEHMERINNEN FÜR ONLINE-UMFRAGE GESUCHT

Wir möchten wissen, wie gut Frauen mit PCOS von Ihren Frauenärzten/innen betreut werden. Dazu ist IHRE Meinung gefragt! Auch Frauen die sich von den Symptomen angesprochen fühlen, aber noch keine Diagnose erhalten haben, werden gesucht!

Sie leisten einen wichtigen Beitrag zur Verbesserung der Betreuung und haben die Möglichkeit, eine Auswertung der Daten zu erhalten.

Die Umfrage dauert ca. 15-20 Minuten. Alle Daten werden anonym erfasst.

https://is.gd/pcos_study
