# Supplementary material for: Unmet clinical needs in women with polycystic ovary syndrome regarding chronic non-communicable diseases: A cross‑sectional study
Source: Arch Gynecol Obstet. 2026 Jan 8;313(1):21. doi: 10.1007/s00404-025-08287-x (PMC12783285; doi:10.1007/s00404-025-08287-x)
Supplement: Supplementary file 2 — Supplementary file2 (PDF 60 kb) [file 404_2025_8287_MOESM2_ESM.pdf]

Kanton Bern
Canton de Berne

Gesundheits-, Sozial- und Integrationsdirektion
Kantonale Ethikkommission für die Forschung

Murtenstrasse 31
3010 Bern
Bern
+41 31 633 70 70 (Telefon)
+41 31 633 70 71 (Telefax)
info.kek.kapa@be.ch
www.be.ch/gsi

Dorothy Pfiffner
+41 31 633 70 77
dorothy.pfiffner@be.ch

GSI-KEK, Murtenstrasse 31, 3010 Bern

Julia Estermann
Muriweid 4
6207 Nottwil

Zuständigkeitsabklärung

BASEC-Nr: Req-2020-00801 **Eingangsdatum:** 02/07/2020

Titel: Versorgungssituation und -bedarf bei Frauen mit PCOS

Ergebnis der Zuständigkeitsabklärung

☒ **Nicht zuständig**, d.h. das Vorhaben ist nicht bewilligungspflichtig
Begründung: Das Vorhaben fällt nicht unter das Humanforschungsgesetz, Art. 2, Abs. 1

☐ **Zuständig**: Bewilligung gemäss Humanforschungsgesetz, Art. 2, Abs. 1 **notwendig**.
Bitte reichen Sie der KEK ein Gesuch gemäss www.swissethics.ch ein

Gebühren: CHF 200.-- (Tarifcode 6.0)
Rechnung folgt

Datum/Ort: 08.07.2020/Bern

i.V.

Prof. Dr. med. Christian Seiler
Präsident

Dr. sc. nat. Dorothy Pfiffner
Leiterin wissenschaftliches Sekretariat
