# Supplementary material for: Unmet clinical needs in women with polycystic ovary syndrome regarding chronic non-communicable diseases: A cross‑sectional study
Source: Arch Gynecol Obstet. 2026 Jan 8;313(1):21. doi: 10.1007/s00404-025-08287-x (PMC12783285; doi:10.1007/s00404-025-08287-x)
Supplement: Supplementary file 4 — Supplementary file4 (DOCX 15 kb) [file 404_2025_8287_MOESM4_ESM.docx]

**Table S_1** Screenings recommended according to ESHRE guidelines 2018 / 2023 in women with PCOS.

| **Screening parameter** | **2018** | **Time** | **2023** | **Time** |
| --- | --- | --- | --- | --- |
| Weight monitoring | x | Each visit, at minimum every 6–12 months | x | Each visit, at minimum every 6–12 months |
| Blood pressure | x | Annually | x | Annually |
| Glycaemic status | x | Baseline, then every 1–3 years | x | Baseline, then every 1–3 years |
| Cardiovascular risk factors | x | At least once (including obesity, cigarette smoking, dyslipidaemia, hypertension, impaired glucose tolerance, lack of physical activity) | x | At least once (including obesity, cigarette smoking, dyslipidaemia, hypertension, impaired glucose tolerance, lack of physical activity) |
| Fasting lipid profile | x | If overweight | x | Routine screening at diagnosis |
| Oral glucose tolerance test (OGTT) | x | Baseline in high-risk women.  Planning a pregnancy or seeking fertility treatment, in pregnancy at 24–28 weeks gestation, if not done preconception: <20 weeks gestation | x | Baseline in all woman regardless of BMI. Planning a pregnancy or seeking fertility treatment, in pregnancy at 24–28 weeks gestation, if not done preconception: offer at first prenatal visit |
| Obstructive sleep apnoea (OSA) | x | Whenever related symptoms are present (snoring, waking unrefreshed,  daytime sleepiness) | x | Whenever related symptoms are present (snoring, waking unrefreshed,  daytime sleepiness) |
| Emotional well-being (screening for anxiety and depressive symptoms, psychosexual dysfunction, body image and eating disorders) | x | Routine screening at diagnosis | x | Routine screening at diagnosis |

Differences are characterized by underlining.
